# Molecular Interplay Between Plant Proteins and Polyphenols: pH as a Switch for Structural and Functional Assembly

**DOI:** 10.3390/foods14233991

**Published:** 2025-11-21

**Authors:** Havva Aktaş, Arkadiusz Szpicer, Barbara Strojny-Cieślak, Wojciech Borucki, Ute Schweiggert-Weisz, Marcin A. Kurek

**Affiliations:** 1 Department of Technique and Food Development, Institute of Human Nutrition Sciences, Warsaw University of Life Sciences (WULS-SGGW), 02-776 Warsaw, Poland; havva_aktas@sggw.edu.pl (H.A.); arkadiusz_szpicer@sggw.edu.pl (A.S.); 2 School of Life Sciences, Plant Proteins and Nutrition, Technical University of Munich, 85354 Freising, Germany; 3 Department of Nanobiotechnology, Institute of Biology, Warsaw University of Life Sciences, 02-776 Warsaw, Poland; 4 Faculty of Agriculture and Biology, Department of Botany, Warsaw University of Life Sciences, Nowoursynowska 159, 02-776 Warsaw, Poland; wojciech_borucki@sggw.edu.pl

**Keywords:** protein–polyphenol complexes, anthocyanins, plant proteins, pH effects, structural transitions, antioxidant functionality

## Abstract

Understanding how plant proteins interact with polyphenols under different pH conditions is key to unlocking the full functional potential of natural ingredients in food systems. This study investigates the pH-dependent binding mechanisms and structural transformations of three underutilized plant proteins: mustard protein concentrate (MP), primrose protein meal (PP), and sunflower meal protein isolate (SMP) in complexation with red cabbage polyphenols (RC) using spectroscopic and microscopic techniques, we show that alkaline conditions (pH 7–9) enhance anthocyanin binding, driven by hydrogen bonding and hydrophobic interactions, particularly in PP and SMP. However, this increased binding is accompanied by greater protein unfolding and aggregation, which affects solubility and colloidal behavior. PP9 demonstrated the strongest antioxidant activity, while MP3 retained anthocyanin stability in acidic conditions. Emulsification and foaming properties varied across proteins and pH: PP showed the highest emulsification at acidic pH, MP had superior emulsion stability at alkaline pH, and SMP maintained performance across all conditions. CLSM imaging confirmed that SMP-based emulsions were the most structurally stable. These findings provide molecular insight into how pH governs the assembly, stability, and functionality of protein–polyphenol complexes, paving the way for the rational design of next-generation plant-based food formulations.

## 1. Introduction

The global shift toward more sustainable and functional food systems has grown interest in plant-based proteins and bioactive polyphenols. Plant proteins produced from mustard seed meal, evening primrose meal, and sunflower seed meal are gaining popularity due to their nutritional value, low environmental impact, and adaptability in food compositions. These proteins exhibit a range of functional properties, including emulsification, foaming, and gelation, which are influenced by their amino acid composition, molecular weight distribution, and structural flexibility [1,2,3]. Polyphenols, such as those found in red cabbage, are essential for their antioxidant effects and pH-responsive chromophore activity, benefiting both health improvement and color preservation. However, the functional efficacy of these components is directly related to their interactions, which are influenced by environmental conditions, including pH [4]. Despite their potential, challenges remain in fully harnessing these interactions, such as poor solubility of plant proteins, pH-induced degradation of polyphenols, and inconsistent binding stability within complex matrices.

Demand for clean-label, nutrient-dense foods is driving plant protein–polyphenol systems’ development [5]. Although soy, pea, and wheat gluten proteins have been thoroughly researched, underused sources like MP, PP, and SMP have not received as much attention despite having distinct functional profiles. For example, MP contains cruciferin, which aids in thermal stability, but SMP is abundant in helianthinin, a globulin with a high emulsifying capability [6,7]. Conversely, polyphenols are increasingly utilized in functional meals, although they have stability problems in neutral to alkaline environments [8]. General protein–polyphenol interaction mechanisms, including electrostatic interactions, hydrogen bonding, and hydrophobic forces, as well as their pH dependency, have been clarified by recent studies [9]. However, there are gaps in our knowledge of how different proteins interact with polyphenols across pH ranges because the majority of research focuses on animal proteins or common plant sources. The struggle between stability and functionality is crucial in the food industry. For example, alkaline pH can improve polyphenol binding by deprotonating it, but it can also cause protein aggregation or anthocyanin breakdown, which affects antioxidant retention or emulsification [10]. Furthermore, it can be difficult to achieve regular interactions due to the inherent heterogeneity of plant proteins, such as the low molecular weight profile of PP or the structural diversity of SMP [11]. Current approaches, including encapsulation or protein modification, frequently change nutritional integrity or are not practical. These drawbacks highlight the necessity of a methodical assessment of pH-dependent interactions between polyphenols and poorly understood plant proteins to maximize their synergistic potential [12].

While extensive studies have focused on widely used legume proteins such as soy and pea in polyphenol interaction and delivery systems, less attention has been paid to underutilized oilseed-derived proteins such as those from mustard, sunflower, and evening primrose. These proteins are primarily by-products of the oil industry and represent promising, sustainable alternatives with distinct structural and functional characteristics compared to traditional plant proteins. Their unique amino acid composition, protein fractions, and surface properties may influence their binding mechanisms with polyphenols and contribute to novel stabilization behaviors. This study pioneers the comparative characterization of these three unconventional proteins in complexation with red cabbage-derived polyphenols across varying pH conditions, aiming to uncover new insights into protein–polyphenol interactions beyond conventional sources and support the valorization of agri-food side streams [13].

In the present study, MP, PP, and SMP were chosen for their different biochemical compositions and functional properties, specifically their emulsifying capacity, solubility behavior, and cysteine-rich domains. These proteins are promising, yet underexplored, byproducts of oilseed processing with the potential for value-added uses in polyphenol delivery systems. Their selection provides a comparative analysis of structure-function connections in protein–polyphenol interactions at various pH levels.

**Figure 1:**
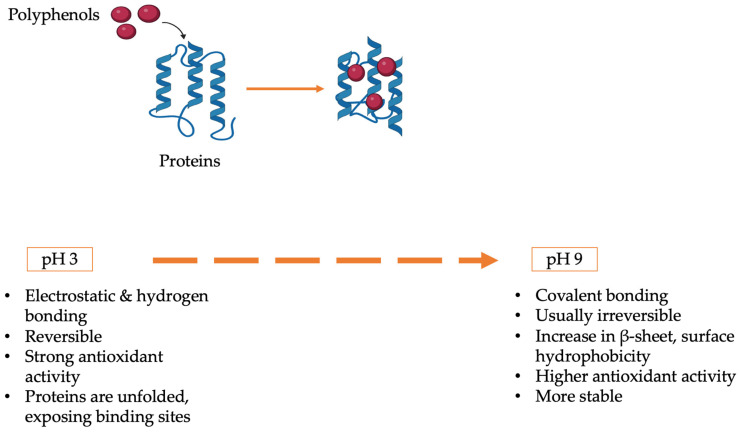
Conceptual model illustrating pH-dependent mechanisms in protein–polyphenol interactions and their impact on functional properties.

This study addresses these gaps by examining the pH-dependent structural and functional interactions of MP, PP, and SMP with RC. The study aims to determine the optimal pH ranges for stabilizing functional complexes, assess binding mechanisms and their impact on antioxidant activity, emulsification, and thermal stability, and characterize conformational changes in proteins and polyphenols under acidic, neutral, and alkaline conditions. This study compares three plant proteins, providing information about their benefits and drawbacks through the application of the most recent analytical techniques, including FT-IR, fluorescence spectroscopy, DSC, and CLSM. To promote sustainable food innovation, the results aim to inform the logical design of plant-based protein–polyphenol systems for use, including pH-stable emulsions, antioxidant-enriched beverages, and thermally resistant nutraceuticals (Figure 1).

## 2. Materials and Methods

### 2.1. Materials

The mustard protein, primrose protein, and sunflower meal protein used in this study were produced at the university facilities. The raw materials for the protein extractions were commercially sourced. Ground mustard seeds were purchased from AROMAT (Radom, Poland) and labeled as a 100% white mustard meal. Sunflower meal was obtained from MAR-ROL (Koźmin Wlkp., Poland), labeled as a feed material with a minimum protein content of 35%. Evening primrose meal was sourced from SAMFARM (Międzyrzec Podlaski, Poland). The proximate composition of the protein materials used in this study was obtained from the literature. For SMP, the composition included 3.47 ± 0.13% moisture, 0.32 ± 0.001% fat, and 1.84 ± 0.09% ash. Regarding MP, it had 14.80 ± 0.2% fiber, 15.67 ± 0.6% fat, 1.23 ± 0.3% ash, and 9.73 ± 0.6% moisture. PP meal was reported to contain 10.34–11.91% moisture, 5.26–5.83% fat, and 4.62–6.01% ash [3,14,15]. Red cabbage powder, used as a source of anthocyanin, was prepared at the university by drying and grinding fresh red cabbage (Brassica oleracea var. capitata f. rubra) obtained from a local market. Soybean oil was purchased from Dary Natury (Grodzisk, Poland). Unless otherwise stated, all other chemicals used in the experiments were of analytical grade and purchased from Sigma-Aldrich (St. Louis, MO, USA).

### 2.2. Preparation of Protein and Anthocyanin Samples

The proteins used in this study, including PP, MP, and SMP, were extracted using the alkaline extraction (AE) method as described by Aktaş et al. (2024) [16]. Briefly, the raw materials were mixed with distilled water at a ratio of 1:100 (g/mL), and the pH was adjusted to 9.5 using 1 M NaOH solution. The slurries were incubated in a water bath at constant shaking for 120 min. After incubation, the mixtures were centrifuged at 9000 rpm for 20 min to separate the protein extract. The protein extract was then acidified to its isoelectric point to precipitate the protein. The isoelectric points were set as follows: 4.0 for PP and SMP, and 5.0 for MP. Acidification was achieved using 1 M HCl, based on methodologies from Hadidi et al. (2021) [17], Sarker et al. (2015) [14], and Zaky et al. (2022) [3]. The precipitated proteins were collected by centrifugation at 9000 rpm for 20 min, washed thoroughly with distilled water to remove residual solubles, and neutralized to pH 7.0. The extracted proteins were subsequently freeze-dried using a freeze-dryer (Christ LSC, Berlin, Germany), vacuum-packed, and stored at 4 °C until further analysis.

A 50 milliliter solution of methanol and 1% hydrochloric acid (MeOH:HCl) was prepared and combined with 1 g of lyophilized red cabbage powder. This mixture was subjected to microwave-assisted extraction. The extraction parameters were set to a power of 90 watts for a duration of 5 min. Following the extraction process as described by Aktaş et al. (2024) [16], the solvent was removed by evaporation, leaving the extract dissolved in distilled water. The resultant solution was then filtered to remove any residual particulate matter.

Unless otherwise specified, three independent biological replicates were used for all analyses, with each sample measured in technical duplicate.

### 2.3. Protein Solubility Was Inferred from Turbidity Measurements at Different pH Levels

Protein solubility was determined according to the method described by Ogunwolu et al. (2009) [18], with slight modifications. Briefly, 200 mg of each protein sample was dispersed in 20 mL of deionized water. The pH of the solutions was adjusted to 2, 4, 6, 8, 10, and 12 using either 1 N or 0.1 N HCl or NaOH solutions, as needed. The mixtures were stirred at room temperature (~20 °C) for 30 min using a magnetic stirrer and then centrifuged at 7500 rpm for 15 min. The protein content in the resulting supernatant was quantified using the Bradford assay [19].

### 2.4. Protein Content Determination

The protein content of the samples was analyzed using the Dumas combustion method (TruMac N, Leco Instruments, Mönchengladbach, Germany). The nitrogen content was converted to protein using the average nitrogen-to-protein conversion factor of N × 6.25. All analyses were conducted in duplicate and followed the AOAC Official Methods (AOAC, 2003) [20].

### 2.5. Sodium Dodecyl Sulfate-Polyacrylamide Gel Electrophoresis (SDS-PAGE)

The molecular weight distribution of the protein samples and their coacervates was analyzed using SDS-PAGE under reducing conditions. A 4–20% Mini-PROTEAN^®^ TGX™ Precast Protein Gel (Bio-Rad, Hercules, CA, USA) and Precision Plus Protein™ Dual Color Standards (10–250 kDa) were used as molecular weight markers. Samples were prepared by dissolving them in Laemmli buffer (0.5 M Tris-HCl, pH 6.8) containing 75 mM 1,4-dithiothreitol, 3% SDS, 10% glycerol, and 0.01% bromophenol blue. The protein concentration was adjusted to 2–3 mg/mL, verified using the Lowry assay.

Samples were homogenized at 10,000 rpm for 2 min using a T18 digital homogenizer equipped with an S18N-10G dispersing tool. The homogenized samples were denatured by heating at 95 °C for 5 min and then centrifuged at 13,000 rpm for 15 min at 4 °C to remove particulates. A 10 µL aliquot of the supernatant was loaded onto the gel for electrophoresis.

Electrophoresis was performed at a constant voltage of 95–120 V in a running buffer composed of 0.25 M Tris, 1.92 M glycine, and 0.5% SDS. After electrophoresis, the gels were rinsed with MilliQ water and stained for 1 h with 0.005% Coomassie Brilliant Blue G250 in a solution of 50% methanol, 40% water, and 10% glacial acetic acid. Gels were destained in 10% methanol and 7.5% glacial acetic acid for 24 h. Protein bands were visualized and analyzed using Image Lab™ Software with the Molecular Imager Gel Doc™ XR1 system (Bio-Rad, Hercules, CA, USA).

### 2.6. Preparation of Protein–Polyphenol Complexes

MP, PP, and SMP were dispersed in distilled water to prepare 1% (*w*/*v*) protein solutions. Protein and red cabbage anthocyanin (RC) complexes were then formed by mixing the protein solutions (1%, *w*/*v*) with RC at a final concentration of 0.3% (*w*/*v*), following the method described by Li et al. (2020) [21]. The pH of the complexes was adjusted to 3, 7, and 9, resulting in complexes labeled MP3, MP7, MP9, PP3, PP7, PP9, SMP3, SMP7, and SMP9.

To remove unbound polyphenols, the complexes were subjected to extensive dialysis using Spectra/Por^®^ 6 Dialysis Membrane Tubing (10 kDa MWCO, nominal flat width: 45 mm, diameter: 29 mm, vol/length ratio: 6.4 mL/cm, Spectrum Laboratories, Laguna Hills, CA, USA) in water at 4 °C for 48 h. The dialysate water was replaced at least five times to ensure the thorough removal of free polyphenols. The reaction mixtures in the dialysis tubes were freeze-dried using a laboratory freeze-dryer (Christ LSC, Berlin, Germany) to obtain lyophilized protein–polyphenol complexes for further analysis.

### 2.7. Binding Capacities

#### 2.7.1. Polyphenol Binding Capacity Was Quantified Using the Folin–Ciocalteu Method

The polyphenol binding equivalents were measured using the Folin–Ciocalteu reducing capacity method using a Photometer Specord 210 Plus (Analytik Jena AG, Jena, Germany) [22]. In brief, 100 μL of the PI-polyphenol complexes (1 mg/mL, dissolved in deionized water) was mixed with 1 mL of 0.2 N Folin–Ciocalteu reagent for 5 min, with care taken to avoid exposure to light. It was then incubated for an additional two hours with 0.8 mL of Na_2_CO_3_ at 7.5% (*w*/*v*). After that, measurements of 765 nm absorbance were taken, and the results were expressed as gallic acid conjugate equivalents.

#### 2.7.2. Free Sulfhydryl Group Content Was Determined via Ellman’s Reagent Assay

The free sulfhydryl (SH) group content of protein and protein–polyphenol complexes was determined following the method described by Pi et al. (2023) [13], with slight modifications. Each sample solution (0.4 mL, 0.5 mg/mL) was mixed with 0.6 mL of 0.1 M phosphate-buffered solution (pH 8.0) and 10 µL of Ellman’s reagent (4 mg of DTNB/mL in phosphate buffer). The mixture was allowed to react at 37 °C for 20 min.

The absorbance of the resulting solution was measured at 412 nm using a Photometer Specord 210 Plus (Analytik Jena AG, Jena, Germany). The free SH group content was calculated using the following formula:

(1) $$SH\;\left(\frac{umol}{g}\right)=\;\frac{73.53\;\times A412\;\times \;D}{C}$$

- 73.53 is the conversion factor;
- A_412_ is the absorbance at 412 nm;
- D is the dilution factor;
- C is the sample concentration (mg/mL).

The results were expressed as micromoles of free SH groups per gram of protein.

### 2.8. Fluorescence Spectroscopy Was Used to Analyze Protein Conformational Changes

An Infinite F200 Pro automated plate reader (Tecan, Männedorf, Switzerland) was used to record the fluorescence spectra of the samples at 25 °C [23]. The concentrations of the three proteins were 0.5 mg/mL, and the concentrations of the complexes were also 0.5 mg/mL, respectively. The emission wavelength was measured in the range of 300 nm to 500 nm at an excitation wavelength of 280 nm. The slit width for excitation and emission was set at 5.0 nm. The emission wavelength step is set to 2 nm.

### 2.9. Surface Hydrophobicity Was Assessed Using ANS Fluorescence Probe

The surface hydrophobicity of the protein and protein–polyphenol complexes was determined using the 1-aniline-8-naphthalenesulfonic acid (ANS) fluorescent probe method, as modified by Wang et al. (2024) [24]. Briefly, 30 μL of an eight mM ANS solution was added to 3 mL of each sample at a concentration of 0.125 mg/mL. The fluorescence intensity was measured using an Infinite F200 Pro automated plate reader (Tecan, Männedorf, Switzerland) with the following settings: 390 nm excitation wavelength, 470 nm emission wavelength, and a 5 nm slit width. The initial slope of the fluorescence intensity versus sample concentration curve was calculated to express the surface hydrophobicity (H_0_) of each sample.

### 2.10. Turbidity

The turbidity of proteins and their complexes at three different pH values (3.0, 7.0, and 9.0) was measured to assess their solubility [25]. Samples were dissolved in distilled water at a concentration of 1 mg/mL. The pH of the solutions was adjusted to 3.0, 7.0, and 9.0 using 0.1 N HCl or NaOH. The absorbance of the solutions was recorded at 600 nm using a Photometer Specord 210 Plus (Analytik Jena AG, Jena, Germany). Measurements were performed in triplicate to ensure reproducibility.

### 2.11. Particle Size and ζ-Potential Were Analyzed Using Dynamic Light Scattering (DLS)

Zetasizer Nano ZS90 (Malvern Instruments, Worcestershire, UK) [25, 26]. Complexes and protein samples were dispersed in 0.01 M Tris buffer (pH 8.4 ± 0.02) at 0.1% (*w*/*v*), agitated at room temperature for 30 min, and filtered through Whatman No. 1 filter paper. The filtrates were analyzed at 25 °C, with triplicate measurements. The particle size of the samples was determined using dynamic light scattering. The preparation was identical to that for zeta potential analysis. The particle size was expressed as the hydrodynamic diameter (DH), and the polydispersity index (PDI) was also reported. Triplicate measurements were conducted for each sample.

(2) $$DH=\frac{KT}{3\pi \eta D}$$

(3) $$PDI={\left(\frac{\sigma }{2\sigma }\right)}^{2}$$

### 2.12. Foaming Properties Were Evaluated Using a Dynamic Foam Analyzer

The foaming properties of the samples were investigated using a Dynamic Foam Analyzer (DFA 100, Krüss GmbH, Hamburg, Germany), as described by Brückner-Gühmann et al. (2018) [27]. A 50 mL protein solution at 1% concentration was poured into a glass column with a diameter of 40 mm (CY4572 prism, Infineon Technologies, San Jose, CA, USA). Air was introduced at a flow rate of 0.3 L/min through an internal source and dispersed using a sparging sample holder (SH4511). The height of foaming and height decay were recorded at five frames per second (fps), while the structural foaming and structural decay were recorded at one fps. Foaming ability was evaluated using the foaming speed (K_f_, mm/min), which reflects the rate of adsorption and stabilization of the newly formed interfacial area.

### 2.13. Emulsion Activity and Stability Were Determined via Oil–Water Emulsification and Centrifugation

The emulsifying properties of protein–polyphenol complexes were evaluated using the procedure described by Wang et al. (2024) [24]. The complexes were dispersed in deionized water and mixed with soybean oil in a 4:1 (*v*/*v*) ratio to form emulsions. The emulsification process was carried out using a homogenizer (T18, IKA-Werke GmbH & Co. KG, Staufen, Germany) at 12,000 rpm for 2 min.

Following homogenization, 50 μL of the emulsion was thoroughly mixed with 4.95 mL of 1% (*w*/*v*) sodium dodecyl sulfate (SDS) solution. The absorbance of the resulting mixtures at 500 nm was measured immediately after mixing (time 0) and again after 30 min. The measurements provided insights into the emulsification activity and stability of the protein–polyphenol complexes. The emulsification activity index (EAI) and emulsification stability index (ESI) were calculated as follows:

(4) $$EAI\;\left(\frac{m^{2}}{g}\right)=\;\frac{4.606\;\times A_{0}\;}{C\;\times \;\left(1\;-\;\varphi \right)\times \;10^{4}\;}\;\times \;D\;$$

(5) $$ESI\;\left(\%\right)=\frac{A_{30}}{A_{0}}\times 100$$

where *A*_0_, *D*, *C*, *φ*, and *A*_30_ are the absorbances of the samples at 500 nm, the dilution factor, the initial protein concentration (g/mL), the volume fraction of oil, and the absorbances of the samples at 500 nm after mixing for 30 min, respectively.

### 2.14. Antioxidant Activity Was Evaluated Using ABTS and DPPH Radical Scavenging Assays

The methods employed to neutralize the activities of the samples against ABTS and DPPH radicals were according to Floegel et al. (2011) [28], with modifications. The results were expressed in umol Trolox equivalents per g protein.

#### 2.14.1. Measurement of ABTS (2,2′-Azino-bis (3-ethylbenzothiazoline-6-sulfonic acid))

The ABTS free radical scavenging capacity of the samples was determined according to the method described using the Folch method. A 7 mmol/L ABTS radical solution was produced by mixing ABTS with 2.45 mmol/L potassium persulfate solution and incubating the mixture in the dark for 12–16 h. The absorbance of the solution was adjusted to 0.70 ± 0.02 at 734 nm by diluting the ABTS solution with 150 mM phosphate-buffered saline (pH 7.4).

For the assay, 0.1 mL of the sample solution was mixed with 1.9 mL of the ABTS radical solution, and the absorbance was recorded at 734 nm for 6 min using a UV–vis spectrophotometer.

(6) $$Inhibition\;\left(\%\right)={((A}_{blank}-A_{sample})/A_{ABTS})\times 100$$

#### 2.14.2. Measurement of DPPH (2,2-Diphenyl-1-(2,4,6-trinitrophenyl) hydrazyl)

The antioxidant activity of the samples was measured using the DPPH assay. A solution of 0.06 mmol/L DPPH was prepared in methanol and adjusted to an absorbance of 0.70 ± 0.02 at 517 nm. The solution was protected from light during the experiment.

For the analysis, 50 µL of the sample or solvent (blank) was added to a tube containing 1.95 mL of the DPPH solution, thoroughly mixed, and incubated in the dark for 30 min. The absorbance was measured at 517 nm using a spectrophotometer.

(7) $$Inhibition\;\left(\%\right)={((A}_{blank}-A_{sample})/A_{blank})\times 100$$

### 2.15. Thermal Properties Were Characterized by Differential Scanning Calorimetry (DSC)

The thermal characteristics of the protein isolates and complexes were analyzed using. DSC (Mettler Toledo, Schwerzenbach, Switzerland) following the method of Zhao et al. (2019) [29]. The instrument was calibrated using pure indium and zinc standards. Approximately 5.0 ± 0.1 mg of each sample was sealed in a standard 40 µL aluminum pan (ME-51119870) with a matching lid (ME-51119871) using a Mettler Toledo Crucible Sealing Press. The analysis was conducted under a nitrogen atmosphere at a flow rate of 100 cm^3^/min. The temperature was scanned from 10 °C to 230 °C at a rate of 10 °C/min. The onset, maximum, and end temperatures, as well as the enthalpy changes (ΔH, J/g), were analyzed using STARe software.

### 2.16. Molecular Structure Was Analyzed Using Fourier-Transform Infrared (FT-IR) Spectroscopy

FT-IR analysis of protein isolates and their complexes was performed using an FT-IR spectrometer (Model Nicolet iS10, Thermo Fisher Scientific, Waltham, MA, USA). Spectra were recorded in the wavelength range of 4000–400 cm^−1^. Thermo Fisher Scientific’s OMNIC software was used to analyze the spectra [16].

### 2.17. Microstructure of Complexes Was Visualized by Confocal Laser Scanning Microscopy (CLSM)

The microstructure of the emulsions was assessed using a confocal laser scanning microscope (Leica Microsystems CMS, Wetzlar, Germany) [30]. The emulsions were created by blending a 3% (*w*/*w*) oil phase (soybean oil) and a 97% (*w*/*w*) aqueous phase for 2 min using a T18 digital Ultra Turrax (IKA, Staufen, Germany). Nile blue in an isopropanol solution (1 mg/mL) was used for staining. The excitation wavelengths were set to 488 nm and 633 nm.

### 2.18. Statistical Analysis

Significant differences between mean values, assessed by one-way analysis of variance, were denoted by a *p*-value ≤ 0.05. (ANOVA). At *p* ≤ 0.05, the Tukey post hoc test was employed to identify statistically significant results. Statistica 13.3 software (StatSoft, Inc., Tulsa, OK, USA) was used for this study.

## 3. Results and Discussion

### 3.1. Protein Content and Solubility

The Dumas combustion method was used to determine the SMP, MP, and PP protein content. The results were expressed as a percentage of protein content using the nitrogen-to-protein conversion factor (N × 6.25).

The Dumas combustion technique was used to measure the SMP, MP, and PP protein contents. SMP has the most significant protein content (89.60 ± 1.21%), followed by MP (67.63 ± 0.61%) and PP (49.74 ± 0.69%). There were significant changes in protein content amongst the samples (*p* ≤ 0.05). The observed protein concentration differences across the three samples are impacted by extraction efficiency, raw material composition, and impurity removal. Due to its higher purity, sunflower meal protein isolate may be a suitable choice for further functional research, such as emulsion activity tests and polyphenol binding assays. Higher protein purity enhances polyphenol binding by providing more accessible binding sites and minimizing interference from non-protein substances such as fibers, lipids, or carbohydrates. On the other hand, mustard and primrose protein concentrates, despite having reduced protein levels, may possess distinct functional capabilities due to the presence of bioactive substances, such as phenolics or lipids, that remain attached to the proteins [3, 6, 17].

**Figure 2:**
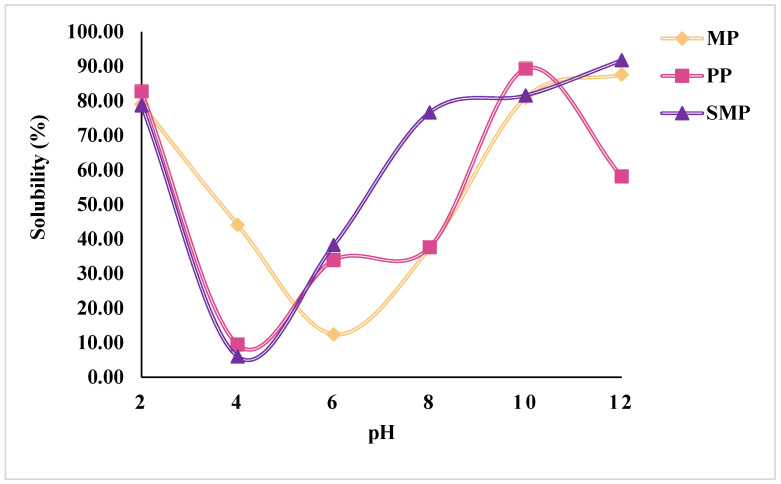
pH-dependent solubility of protein isolates.

The solubility profiles of SMP, MP, and PP as a function of pH are shown in Figure 2. The solubility of all three protein isolates exhibited a characteristic U-shaped curve, with the lowest solubility observed in the acidic pH range around pH 4–6, corresponding to their isoelectric points (pI). At this pH, electrostatic repulsion is minimized, leading to increased aggregation and precipitation of proteins, a common phenomenon in plant-based proteins [31]. At alkaline pH (pH 10–12), protein solubility significantly increased, with SMP reaching the highest solubility at 91.76% at pH 12, followed by MP (87.64%) and PP (58.10%). This trend is attributed to increased electrostatic repulsion and protein unfolding, leading to higher water-protein interactions [32]. Similarly, at highly acidic pH (pH 2), proteins demonstrated high solubility (~79–83%) due to the protonation of amino acid residues, which enhances repulsion and solubilization [33].

Among the three proteins, SMP exhibited the highest overall solubility across pH levels, especially in the alkaline range, indicating superior dispersibility. PP displayed the lowest solubility at pH 4 (9.44%), suggesting strong aggregation tendencies. Compared to MP and SMP, PP retained lower solubility at higher pH values. This behavior may reflect differences in its protein profile or physicochemical properties. The observed solubility trends highlight the pH-dependent behavior of plant proteins, providing critical insights into their potential applications in food formulations that require high solubility, such as emulsions or beverages. The differences in solubility behavior among the proteins emphasize the role of their inherent protein structure, molecular interactions (including hydrogen bonding, hydrophobic interactions, and disulfide linkages), and extraction conditions (e.g., pH shifts, ionic strength, and precipitation steps), all of which influence protein unfolding, dispersion, and water affinity.

### 3.2. SDS-PAGE Analysis

**Figure 3:**
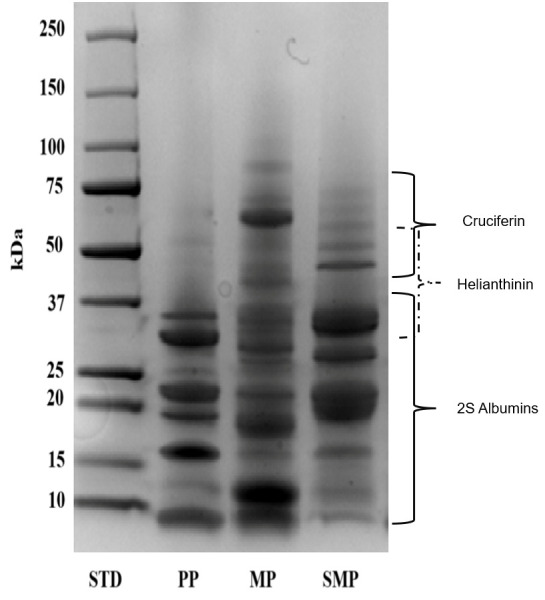
SDS-PAGE profile of protein samples.

The molecular weight distribution of the protein isolates, including PP, MP, and SMP, was assessed using SDS-PAGE under reducing conditions (Figure 3). The distinct protein banding patterns highlight the unique structural composition of the proteins derived from different plant sources.

The SDS-PAGE profile of PP exhibited strong bands in the low molecular weight range of 10–37 kDa, which corresponded to seed storage proteins, including albumins. The finding aligns with the reported properties of specific plant proteins. For example, rapeseed has a low-molecular-weight protein called 2S albumin-type napin [34]. However, the preponderance of smaller proteins may limit its ability to emulsify. Larger proteins with amphipathic characteristics, possessing both hydrophobic and hydrophilic regions, are frequently needed for emulsification—the process by which a protein stabilizes an emulsion of water and oil—to coat oil droplets efficiently and prevent coalescence [35]. The capacity of PP proteins to provide stable contact between the water and oil phases may be limited by their reduced size.

Conversely, MP showed a broader range of molecular weights, with the predominant bands ranging from 50 to 75 kDa. These relate to cruciferin, the primary storage protein in mustard seeds. Cruciferin, a 12S globulin-type protein, is known for its diverse functional properties, including gelling and emulsifying capabilities [34]. Furthermore, napin, a minor storage protein, is present when secondary bands are less than 37 kDa. The presence of globular (cruciferin) and smaller (napin) structural proteins suggests a complex interplay of interactions that influences the functional properties of MP. The larger cruciferin molecules may contribute to emulsifying and gelling properties, while the smaller napin molecules might enhance solubility and polyphenol binding [36].

SMP exhibited a highly varied protein profile, with bands ranging widely in molecular weight from 10 to 75 kDa. The main storage protein in sunflower seeds, helianthinin, is notably characterized by strong bands between 37 and 50 kDa [37]. Faint bands at higher molecular weights may indicate inadequate denaturation or protein aggregation, whereas bands below 25 kDa may indicate partial hydrolysis during the extraction process. Aggregation can occur due to various factors, including protein–protein interactions and improper sample handling. Conversely, bands below 25 kDa could indicate partial hydrolysis of the proteins during extraction. Hydrolysis, the breakdown of proteins into smaller peptides, can occur during the extraction process or due to the presence of proteases in the sample [38]. SMP’s wide molecular weight dispersion indicates that its structural heterogeneity may lead to improved functional characteristics, such as polyphenol binding and emulsification.

The observed molecular weight distributions are critical in determining the interactions of these proteins with polyphenols in complex formation and their functional behavior in food systems. The presence of multiple low- and high-molecular-weight proteins may contribute to the enhanced stabilization of emulsions and encapsulation properties in SMP and MP, compared to PP. The findings presented here emphasize the importance of considering the protein composition and molecular weight distribution when evaluating the functional properties and potential applications of plant-based proteins [39]. These three proteins offer unique characteristics that could be exploited in diverse applications, depending on the required functional properties. Further research, including detailed functional characterization and comprehensive analysis of amino acid composition and digestibility, is crucial for a full understanding of the potential of these proteins as sustainable and nutritious alternatives in food systems.

### 3.3. Determination of Binding Abilities

#### 3.3.1. Polyphenol Binding Capacity

**Figure 4:**
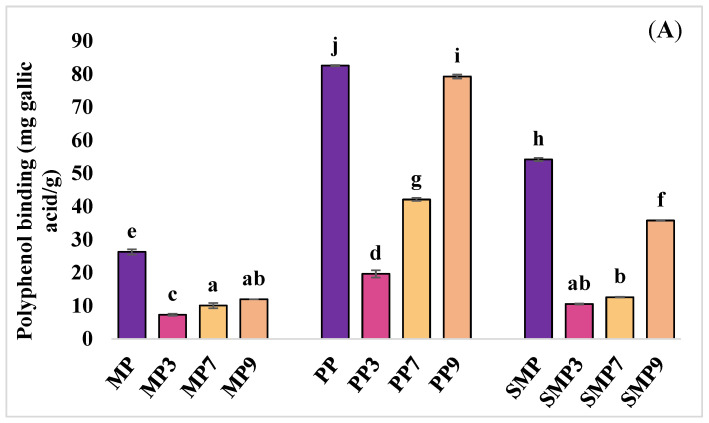

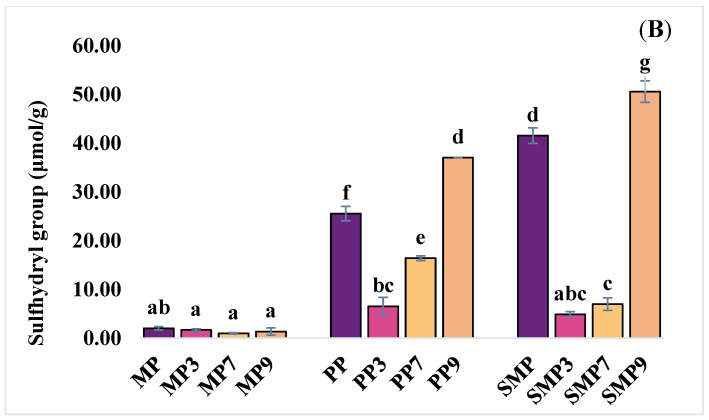
Effect of different protein types on polyphenol binding (**A**) and sulfhydryl group availability (**B**). In the represented data, different superscript letters indicate statistical significance at *p* ≤ 0.05.

The polyphenol binding capacity of the protein isolates and their complexes at different pH values is presented in Figure 4A. The highest polyphenol binding was observed in PP and its complex at pH 9 (PP9), followed by SMP and its complex at the same pH (SMP9). MP and its complexes exhibited significantly lower polyphenol binding capacity compared to PP and SMP. This suggests that the structural properties of PP and SMP may offer more available binding sites for polyphenols from red cabbage to MP.

Polyphenol binding is influenced by the availability of hydroxyl groups in polyphenols, which form hydrogen bonds with carbonyl and amide groups in proteins [9]. The higher binding observed at alkaline pH (pH 7 and 9) suggests that deprotonation of phenolic hydroxyl groups enhances hydrogen bonding and electrostatic interactions with negatively charged protein residues, such as aspartate and glutamate. Kieserling et al. (2024) [11] demonstrated that higher pH levels promote the deprotonation of phenolic hydroxyl groups, enhancing their interaction with protein amino groups. At an acidic pH (pH 3), a significant decrease in polyphenol binding was observed, particularly in MP3 and SMP3, likely due to increased protein aggregation and reduced solubility, which limits the accessibility of binding sites. This aligns with findings from Martins et al. (2023) [40], which described how protein aggregation can influence binding interactions with polyphenols. These findings suggest that the type of protein and pH significantly affect the effectiveness of polyphenol complexation, which could impact their functional properties in food applications.

#### 3.3.2. Sulfhydryl Group

SH group content of the protein isolates and their complexes is shown in Figure 4B. The highest free SH group content was observed in SMP9 (50.61 µmol/g), followed by PP9 (37.06 µmol/g). In contrast, the lowest values were recorded in MP3 (1.69 µmol/g) and MP7 (1.00 µmol/g). The significant increase in free SH content in SMP and PP at higher pH levels suggests that alkaline conditions promote the exposure of buried sulfhydryl groups due to the partial unfolding of proteins, thereby enhancing their reactivity. This study yields similar results to those reported by Guo et al. (2024) [41], who found that polyphenols can interact with protein sulfhydryl groups, thereby reducing the free SH content.

The increase in sulfhydryl content at alkaline pH nine can be attributed to several factors, including a change in protein conformation that exposes more cysteine residues, the disruption of protein-anthocyanin complexes under alkaline conditions, or a combination of both [42, 43]. Overall, the findings demonstrate the interaction between relationships among polyphenols and changes in protein structure. Higher pH levels promote polyphenol binding but also increase the exposure of SH groups, indicating a precise balance between complex formation and protein unfolding. These findings are critical for optimizing protein–polyphenol interactions to enhance the stability and functionality of encapsulated polyphenols in food systems.

### 3.4. Fluorescence Emission Spectroscopy

**Figure 5:**
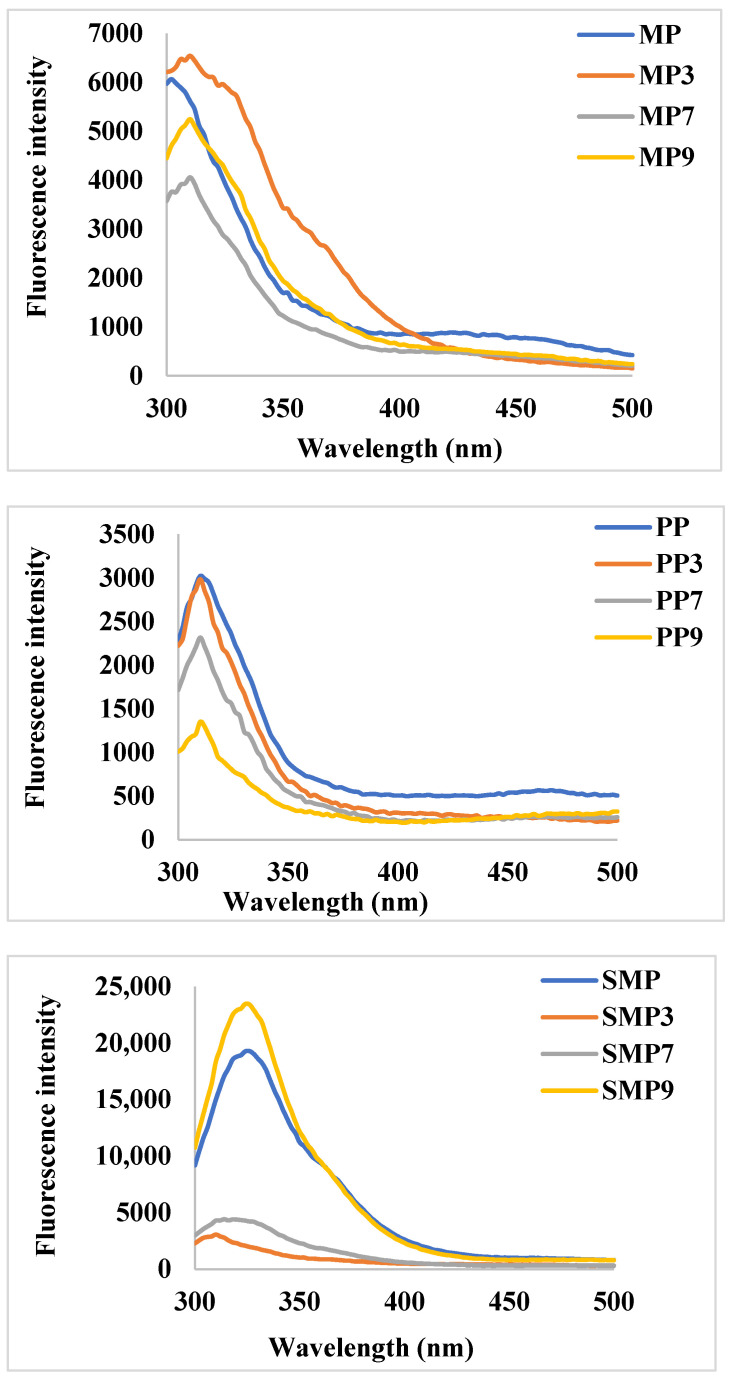
Fluorescence emission spectra of MP, PP, and SMP and their complexes at different pH conditions.

Changes in fluorescence intensity provide insights into structural modifications and complex formations influenced by pH-dependent interactions (Figure 5). The native MP exhibited moderate fluorescence intensity, with a peak around 330–350 nm, characteristic of tryptophan and tyrosine residues. Upon anthocyanin complexation, a significant increase in fluorescence intensity was observed at pH 3 (MP3), suggesting enhanced hydrophobic interactions and partial unfolding of the protein structure. At pH 7 (MP7), a decrease in fluorescence intensity indicated potential quenching effects due to non-covalent interactions between RC and MP. The lowest fluorescence intensity at pH 9 (MP9) suggests stronger electrostatic interactions leading to structural rearrangements and reduced hydrophobic exposure [44].

PP demonstrated lower fluorescence intensity than MP, suggesting a different tertiary structure with fewer exposed fluorophores. Upon complexation, a slight increase in fluorescence intensity was observed at pH 3 (PP3), indicating minor conformational changes. However, at pH 7 and 9, significant quenching effects were noted, with PP9 showing the lowest intensity. This trend suggests a stabilizing effect of polyphenols on PP at alkaline pH, potentially forming stronger hydrogen bonds and electrostatic interactions that shield fluorescent residues from solvent exposure.

SMP exhibited the highest fluorescence intensity among the studied proteins, indicating a more exposed tryptophan environment. Notably, the complex formed at pH 9 (SMP9) displayed the highest intensity, suggesting that polyphenols contributed to enhanced structural rigidity or aggregation, reducing the quenching effect. In contrast, SMP3 and SMP7 exhibited significant fluorescence quenching, which could be attributed to protein conformational changes and stronger hydrogen bonding at lower pH values [45]. These results are consistent with the findings of Dangles & Fenger (2018) [46], who reported that polyphenols can engage in π-stacking interactions and hydrogen bonding with proteins, resulting in alterations in fluorescence properties that depend on the pH.

At acidic pH (3.0), polyphenols predominantly exist in their flavylium cationic form, which carries a positive charge [47]. This positive charge allows for strong electrostatic interactions with negatively charged amino acid residues on the protein surface. The resultant interaction may lead to conformational changes in the protein structure, exposing more hydrophobic regions and tryptophan residues, as evidenced by the increased fluorescence intensity observed in MP3 and the slight increase in PP3 [48].

As the pH increases to neutral (7.0), the equilibrium shifts towards forming quinoidal bases, which are less charged and exhibit a greater capacity for hydrogen bonding. The hydrogen bonding interactions at neutral pH can lead to fluorescence quenching by bringing the RC molecules into proximity with the tryptophan residues, thereby reducing their exposure to the solvent and decreasing fluorescence intensity, as observed in MP7, PP7, and SMP7 [49, 50].

At alkaline pH (9.0), polyphenols predominantly exist in their chalcone or anionic form, leading to different interaction mechanisms with proteins. The pronounced fluorescence quenching in MP9 and PP9 suggests that aggregation-induced effects are at play, possibly due to protein unfolding or self-association. Conversely, the increased fluorescence intensity in SMP9 suggests a stabilizing effect, potentially due to charge repulsion that prevents excessive aggregation, thereby maintaining tryptophan exposure [50]. The observed trends suggest that pH modulation can be strategically employed to control polyphenol–protein interactions, thereby influencing the stability and bioavailability of polyphenols.

### 3.5. Surface Hydrophobic Value (H_o_) of Samples

**Figure 6:**
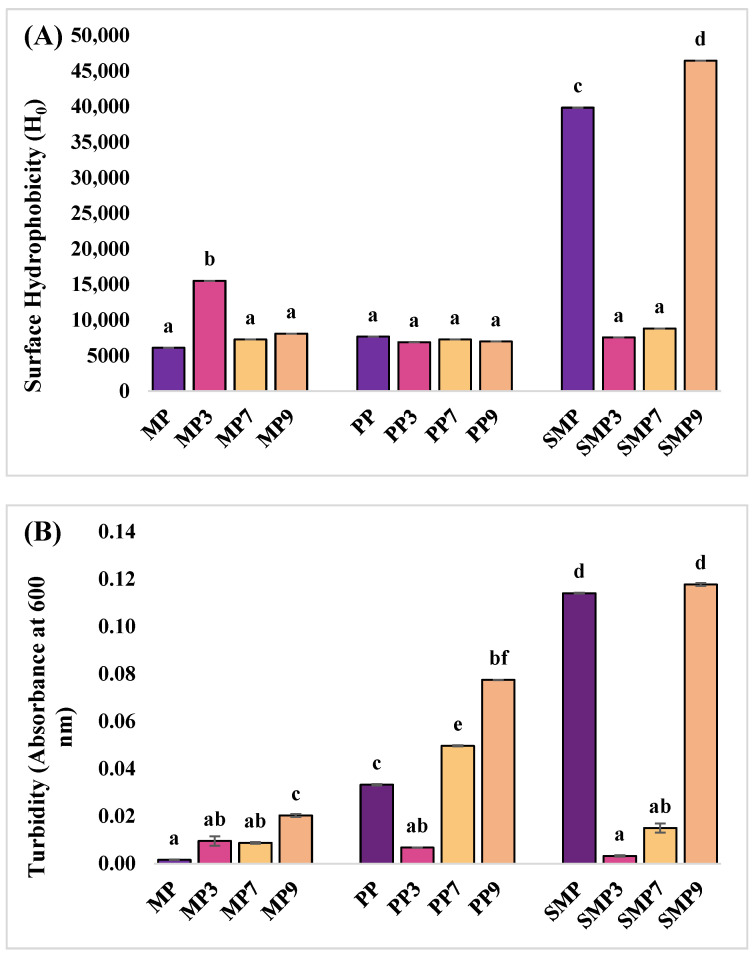

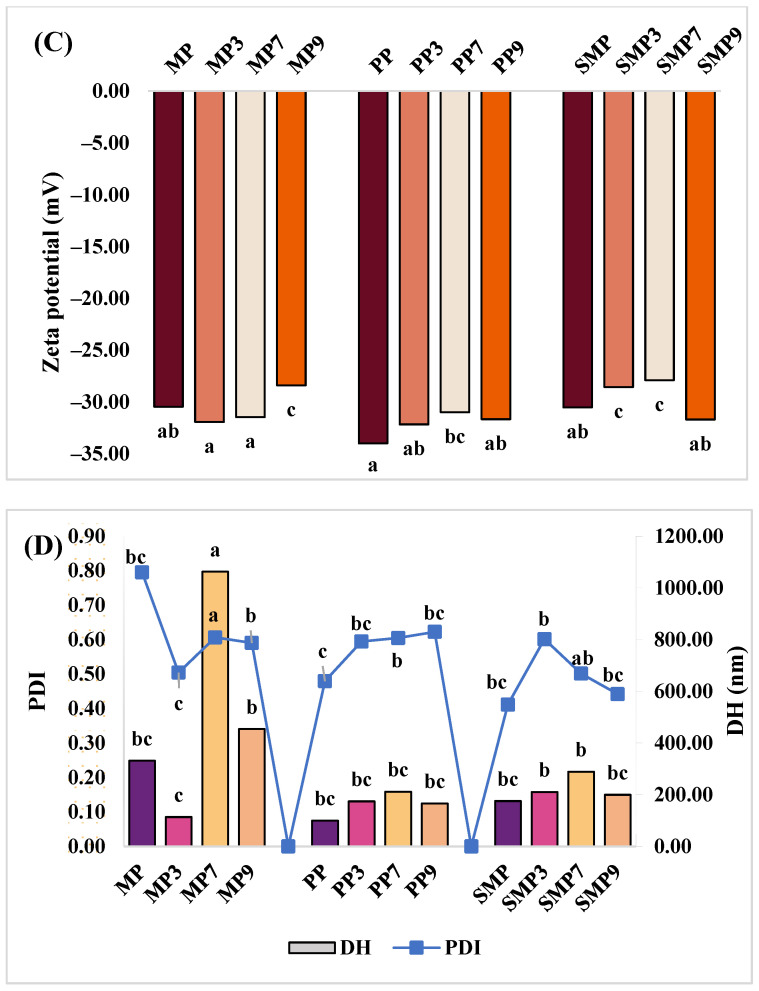
Surface hydrophobicity (**A**), Turbidity (**B**), Zeta potential (**C**), and Particle characteristics—PDI & DH (**D**) of proteins and their complexes at different pH levels. Different superscript letters indicate statistical significance at *p* ≤ 0.05.

Surface hydrophobicity was measured to evaluate the structural changes in proteins upon anthocyanin complexation at different pH conditions (Figure 6A). The native MP exhibited a low surface hydrophobicity value, suggesting limited exposure of hydrophobic residues in its native conformation. Upon anthocyanin binding, a significant increase was observed at pH 3, while hydrophobicity values were lower at pH 7 and 9. The increased hydrophobicity at acidic pH can be attributed to the partial unfolding of MP, exposing hydrophobic residues that interact with the ANS fluorescent probe. PP exhibited moderate hydrophobicity, which decreased slightly upon anthocyanin binding, particularly at pH levels of 3 and 9. The relatively stable hydrophobicity values suggest that anthocyanin binding to PP did not induce significant unfolding or structural exposure of hydrophobic regions. Rashidinejad et al. (2023) [51] reported that specific proteins exhibit minimal changes in hydrophobicity upon interaction with polyphenols, likely due to their intrinsic stability and rigid tertiary structures. At pH 7, the hydrophobicity remained close to the PP value, suggesting that hydrogen bonding was the dominant interaction mechanism at this pH rather than hydrophobic interactions. SMP had the highest hydrophobicity, which significantly decreased at pH levels of 3 and 7. However, at pH 9, SMP9 showed an increase in H_0_, suggesting aggregation or structural rearrangement. Anthocyanin binding significantly reduced hydrophobicity at pH 3 and 7, likely due to changes in protein conformation that led to stronger hydrogen bonding. However, at pH 9, SMP9 displayed the highest hydrophobicity, suggesting aggregation or structural rearrangement. These findings are consistent with the study of Seczyk et al. (2019) [52], who reported that protein–polyphenol interactions are highly dependent on pH and protein type, influencing their physicochemical properties and functional stability.

### 3.6. Turbidity Measurement

The native MP had minimal turbidity, indicating high solubility (Figure 6B). After complexation with polyphenols, MP3 and MP7 remained soluble, but MP9 showed slightly increased turbidity, suggesting reduced solubility at alkaline pH due to charge redistribution and hydrophobic interactions.

PP initially had slightly higher turbidity than MP, indicating lower solubility. PP3 exhibited improved solubility at acidic pH, but turbidity increased at PP7 and PP9, indicating higher aggregation at neutral and alkaline pH levels due to conformational changes. SMP had the highest turbidity, indicating the lowest solubility. SMP3 exhibited improved solubility, but turbidity increased at SMP7 and SMP9, indicating aggregation at higher pH levels.

Overall, the turbidity of all proteins increased at higher pH levels, indicating reduced solubility and increased aggregation. At acidic pH, electrostatic interactions likely enhanced solubility, while at higher pH, hydrophobic interactions promoted aggregation [53]. Differences in turbidity among MP, PP, and SMP suggest that unique structural properties influence complex stability, with SMP showing the most aggregation at alkaline pH.

### 3.7. Analysis of Partial Size Distribution and ζ-Potential

Zeta potential (ZP), PDI, and DH were measured to evaluate the stability, dispersity, and aggregation behavior of protein–polyphenol complexes at different pH levels (Figure 6C,D). The results indicate that pH-dependent variations in surface charge and particle size influence the colloidal stability of the complexes.

MP exhibited ZP values ranging from −30.50 mV to −31.97 mV (MP3), indicating high stability at acidic pH [54]. However, at pH 9, MP9 showed a less negative ZP (−28.43 mV), suggesting a reduction in electrostatic repulsion, which could promote aggregation. This trend is supported by Lan et al. (2020) [55], who reported that reduced negative charge at alkaline pH weakens electrostatic stability, leading to larger aggregates. MP7 had the highest DH (1062 nm), indicating significant aggregation, while MP3 had the smallest DH (114 nm), suggesting better dispersion at acidic pH. The high PDI values for MP (0.79) and MP7 (0.61) indicate broad size distributions, while the lower PDI at MP3 (0.50) suggests improved homogeneity.

PP showed the most negative ZP (−34.03 mV), indicating strong electrostatic stability, particularly at PP3 (−32.20 mV). While PP9 (−31.70 mV) remained relatively stable, the slight decrease suggests a reduced charge repulsion at higher pH levels. Compared to MP, PP complexes exhibited lower DH values, with PP3 (174 nm) and PP9 (166 nm) showing better dispersion than MP9 (454 nm). The PDI values remained moderate (0.48–0.62), indicating a relatively uniform particle size distribution. These results show that proteins with higher negative ZP might exhibit better dispersion and colloidal stability in polyphenol-rich systems [56].

SMP displayed ZP values ranging from −30.57 mV (SMP) to −31.73 mV (SMP9), indicating stable colloidal properties across pH conditions. While SMP7 had the least negative ZP (−27.93 mV), it maintained stability. This trend suggests that the SMP-anthocyanin complexes may be more stable at higher pH levels due to specific interactions between SMP and polyphenols under alkaline conditions. DH values varied, with SMP7 having the largest particles (289 nm) and SMP3 the smallest (210 nm). SMP9 (200 nm) exhibited better dispersion than MP9 (454 nm), in line with findings by Beaubier et al. (2021) [7] that specific plant proteins maintain stability even at alkaline pH when interacting with polyphenols. The moderate PDI values (0.41–0.60) suggest good uniformity in particle distribution.

Overall, while all proteins maintained stable ZP values, MP showed the most aggregation at neutral pH, PP exhibited the best electrostatic stability, and SMP maintained a relatively balanced profile. The lower DH values at acidic pH indicate better dispersion, while higher values at neutral and alkaline pH suggest increased aggregation [57]. These findings highlight the role of pH in modulating protein–polyphenol interactions, with acidic conditions favoring stability and neutral-to-alkaline conditions promoting aggregation, which is crucial for designing functional food systems.

### 3.8. Determination of Foaming Properties

**Figure 7:**
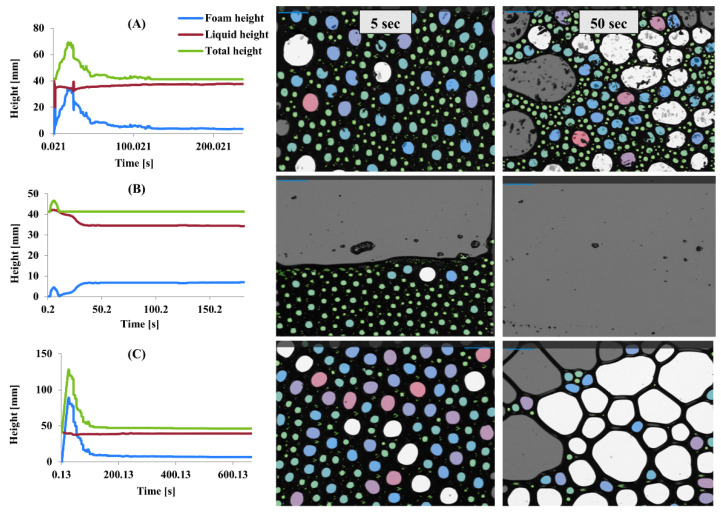
Foaming properties of MP (**A**), PP (**B**), and SMP (**C**) protein isolates and foam structures generated by sparging 0.3 L/min airflow in DFA.

Foaming properties were evaluated by measuring foam height, liquid height, and total height over time for MP, PP, and SMP (Figure 7). Microstructure analysis of bubble size distribution at the 5th and 50th seconds also provided further insight into foam stability and collapse behavior. The differences in foam formation and stability among the protein isolates indicate variations in their interfacial properties and structural behavior.

MP exhibited rapid foam formation, reaching its peak within the first few seconds, followed by a steep decline (Figure 7A). At the 5th second, MP displayed a dense structure with tiny, uniformly distributed bubbles, indicating good initial foaming capacity. However, by the 50th second, coalescence led to more extensive, irregular bubbles, suggesting poor foam stability and rapid collapse. The foaming properties of proteins are influenced by a combination of factors, including their amino acid composition, molecular structure, surface hydrophobicity, and solubility. Proteins that rapidly adsorb at the air–water interface can form foams quickly; however, the stability of these foams depends on the cohesive strength of the protein films. Flexible proteins create foams with larger bubbles and lower stability, whereas proteins that form cohesive films contribute to better foam stability [58]. PP demonstrated the lowest foam height, indicating limited foaming ability (Figure 7B). However, its total height remained stable over time, suggesting better foam retention. Microstructure images show that at the 5th second, PP formed a tightly packed bubble network, though with fewer bubbles than MP and SMP. By the 50th second, most bubbles disappeared, leaving a thin liquid film, confirming that PP foams are more resistant to collapse but have lower aeration. SMP exhibited the highest foam height and better retention over time (Figure 7C). At the 5th second, the microstructure revealed a uniform distribution of tiny bubbles, similar to MP but with greater density. By the 50th second, although some coalescence occurred, SMP maintained a significant number of medium-sized bubbles, indicating superior stability compared to MP.

Proteins with a balanced distribution of hydrophobic and hydrophilic amino acids tend to exhibit better foaming properties, as they can effectively adsorb at the air–water interface and form stable films [59]. The rapid foam formation observed with MP may be related to its ability to quickly adsorb at the interface, while its poor stability may be due to a lack of cohesive strength within the film. PP’s high solubility may contribute to its better foam retention, as it can form a viscous continuous phase that slows down drainage and collapse. SMP’s ability to form strong interfacial films likely stems from its unique structural properties and intermolecular interactions [35].

The stability of protein foams is influenced by pH, temperature, and the protein’s structure. pH affects charge distribution on protein molecules, altering their interactions and foam stability, with the isoelectric point reducing surface charge and impacting adsorption at the air–water interface [60]. Temperature influences protein conformation and solubility, with high temperatures causing denaturation and altering foaming behavior. Additionally, polysaccharides enhance foaming by increasing viscosity and stabilizing the air–water interface. At the same time, other ingredients, such as lipids and proteins, may have positive or negative effects depending on their interactions with the foaming agent [5, 61]. Understanding these factors is essential for optimizing foaming properties in food applications.

### 3.9. Emulsion Activity and Stability

**Figure 8:**
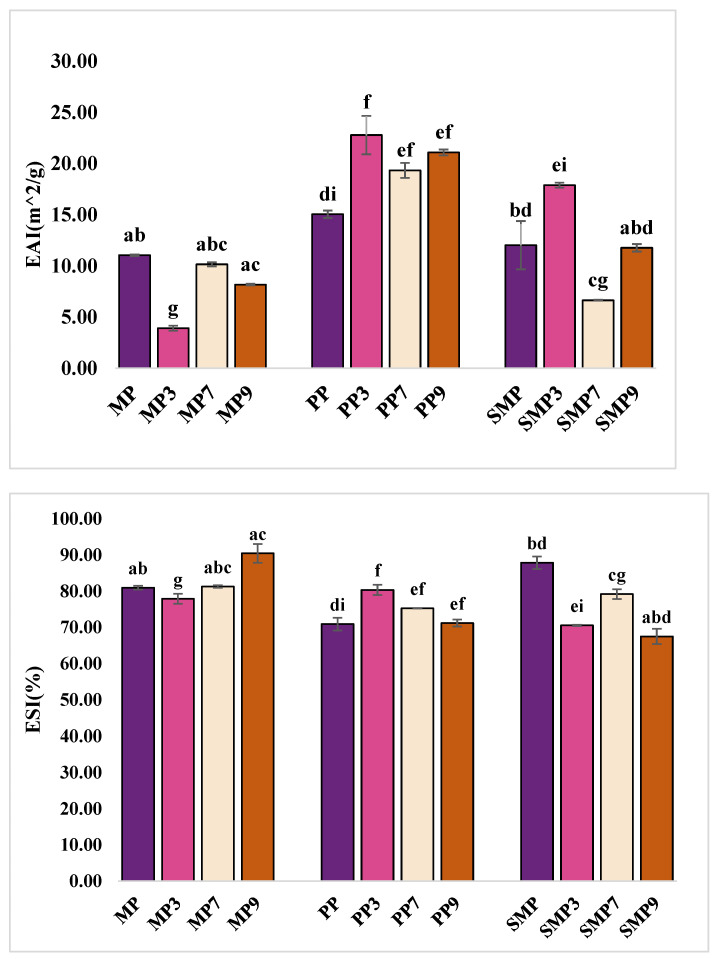
EAI and ESI of MP, PP, and SMP proteins and their complexes. Data are represented as mean ± SD; different superscript letters indicate statistical significance at *p* ≤ 0.05.

EAI and ESI were measured to evaluate the ability of protein–polyphenol complexes to stabilize oil–water emulsions (Figure 8). The results indicate that the emulsification properties of protein–polyphenol complexes depend on their ability to adsorb at the oil–water interface, reduce interfacial tension, and stabilize emulsions by preventing droplet coalescence (Lin et al. 2025) [62].

PP exhibited the highest emulsification activity, with EAI values increasing from 15.06 m^2^/g for PP to 22.80 m^2^/g at pH 3. This suggests that the enhanced interfacial adsorption may be attributed to higher protein solubility and structural flexibility under acidic conditions [63]. The increase in EAI at pH 3 indicates that PP becomes more effective at reducing the interfacial tension between oil and water, resulting in smaller droplet sizes and a larger interfacial area. However, stability decreased at higher pH levels, with ESI values dropping from 80.37% (PP3) to 71.22% (PP9). This decline suggests that while PP forms strong emulsions initially, its interfacial film may weaken over time due to protein aggregation and reduced charge repulsion at alkaline pH [64].

MP exhibited moderate EAI but superior stability at alkaline pH, with MP9 showing the highest ESI with 90.48%, indicating strong resistance to separation. Despite moderate emulsifying activity (8.18 m^2^/g at pH 9), MP likely forms a rigid interfacial film that prevents coalescence, contributing to stability. In contrast, MP3 had the lowest EAI (3.92 m^2^/g), likely due to its restricted flexibility at low pH, which limited adsorption at the oil–water interface. The stability of MP at pH 9 suggests that polyphenol–protein interactions enhance interfacial cross-linking, strengthening the emulsion film. Jahan et al. (2024) [2] also examined the emulsification properties of mustard meal protein and found that pH significantly influenced its emulsifying activity and stability. Their study reported that emulsification activity was highest at alkaline pH (pH 12), where increased solubility and charge repulsion enhanced protein adsorption at the oil–water interface. Conversely, protein aggregation and reduced electrostatic repulsion at lower pH levels led to lower emulsification activity and stability.

SMP demonstrated a balanced emulsification profile, with the highest EAI at pH 3 (17.91 m^2^/g) but reduced stability at pH 9 (67.51%). This suggests that while SMP forms stable emulsions under acidic conditions, aggregation at alkaline pH may lead to destabilization. The high EAI at pH 3 indicates effective interfacial adsorption and droplet formation, while SMP7 had the lowest EAI (6.65 m^2^/g), possibly due to reduced electrostatic repulsion causing flocculation. The decline in ESI at pH 9 suggests a loss of charge repulsion and increased hydrophobic interactions, leading to aggregation and reduced stability. Bojanić et al. (2024) [65] investigated the emulsifying properties of sunflower meal protein, focusing on the influence of protein content and particle size distribution on oil-in-water emulsions. The findings revealed that higher protein content significantly improved the emulsifying capacity of sunflower meal. Additionally, the study observed that particle size distribution played a crucial role in emulsion stability, with smaller particle sizes contributing to more stable emulsions. The studies attributed these improvements to the amphiphilic nature of sunflower proteins, which possess a balanced distribution of hydrophilic and hydrophobic amino acids, facilitating the effective stabilization of oil-in-water emulsions [66]. Polyphenol–protein interactions contributed to emulsion stability by reinforcing interfacial films, though steric hindrance at high polyphenol concentrations may have disrupted emulsification, particularly in SMP9.

The emulsifying behavior of protein–polyphenol complexes across pH values is intricately linked to pH-induced structural modifications in the protein matrices. At acidic pH (3), proteins like PP and SMP displayed improved emulsifying activity, which may be attributed to increased solubility and conformational flexibility that allow better orientation at the oil–water interface. Acidic conditions promote unfolding of protein structures and reduce electrostatic repulsion, facilitating enhanced surface adsorption and interfacial film formation. In contrast, under alkaline conditions (pH 9), MP showed improved emulsion stability despite lower emulsification activity, suggesting that increased sulfhydryl exposure and intermolecular cross-linking enhance the rigidity and strength of the interfacial layer. However, SMP exhibited reduced stability at pH 9, which may stem from excessive aggregation or steric hindrance caused by oxidized polyphenols or rigid protein networks. These observations indicate that pH affects not only the interfacial behavior of proteins but also the nature of protein–polyphenol complexes formed, ultimately dictating their emulsification performance.

### 3.10. Determination of Antioxidant Activities

The antioxidant potential of protein–polyphenol complexes was evaluated using ABTS and DPPH radical scavenging assays, which assess their ability to neutralize free radicals under different pH conditions (Table 1).

The ABTS assay revealed that PP9 exhibited the highest ABTS activity, followed by PP7, whereas SMP3 demonstrated the lowest value. The superior antioxidant activity of PP9 suggests that alkaline conditions favor polyphenol oxidation, leading to the formation of reactive quinonoid structures that enhance the radical scavenging ability [67]. It was observed that protein–polyphenol interactions at alkaline pH enhance electron transfer reactions, thereby improving antioxidant efficiency [68]. M. Li et al. (2021) [69] also examined the interactions between proteins and polyphenols at varying pH levels. They discovered that alkaline environments promote the oxidation of polyphenols, which produce quinones that can interact with proteins. By promoting electron transfer processes, these oxidative changes may enhance antioxidant properties and influence the effectiveness of radical scavenging.

For MP, MP3 showed a higher ABTS activity than MP9, suggesting that acidic conditions better preserve the antioxidant properties of polyphenols at pH 3. Polyphenols remain in their flavylium cation form, which exhibits superior radical scavenging activity compared to its degraded forms at higher pH. The slight reduction in MP9 suggests a possible loss of anthocyanin stability or decreased phenolic availability at alkaline pH [8].

The DPPH radical scavenging activity followed a similar trend as ABTS, with PP9 showing the highest activity, followed by PP7. This suggests alkaline conditions enhance polyphenol reactivity, increasing hydrogen donation potential. MP samples exhibited lower DPPH activity than PP, with MP3 slightly higher than MP9, aligning with findings that polyphenols have greater radical scavenging efficiency at lower pH due to their flavylium cation form. SMP complexes followed a different trend, with SMP3 showing the highest DPPH activity despite poor ABTS performance, suggesting SMP-polyphenol interactions favor hydrogen atom transfer over electron transfer [70]. However, the decline in antioxidant activity at pH 9 indicates possible polyphenol degradation or weaker retention within the SMP matrix.

These variations in antioxidant performance across pH levels can be mechanistically explained by pH-dependent structural and chemical transformations in both proteins and polyphenols. At acidic pH, polyphenols, particularly anthocyanins, exist in their stable flavylium cation form, which has strong radical-scavenging potential. Proteins under low pH are more unfolded, exposing hydrophilic and aromatic residues that may enhance polyphenol binding and stabilize antioxidant capacity. In contrast, alkaline pH promotes deprotonation of phenolic hydroxyl groups, increasing electron-donating ability and facilitating redox cycling and quinone formation. These oxidized forms interact with nucleophilic residues in proteins (e.g., cysteine, lysine), leading to covalent binding and formation of antioxidant-active complexes. However, excessive oxidation or cross-linking can reduce polyphenol availability, as seen in SMP9. Thus, structural transitions such as exposure of sulfhydryl groups, increased surface hydrophobicity, and altered binding conformation—play a critical role in determining whether antioxidant activity is enhanced or diminished under specific pH conditions [71]. These results can guide the optimization of protein–polyphenol systems for functional food applications with antioxidant properties.

### 3.11. Thermal Properties

The thermal behavior of protein–polyphenol complexes was analyzed using DSC, with the results presented in Table 1. The key parameters provide insights into the structural stability and thermal transitions of the complexes under different pH conditions. ΔH represents the energy required for protein unfolding, indicating the extent of molecular interactions and conformational stability. The results show significant differences in ΔH values among the samples, suggesting pH-dependent variations in protein–polyphenol binding and structural rigidity [72].

Among the samples, MP7 exhibited the highest enthalpy change, followed by PP9, whereas MP9 had the lowest. The significantly higher ΔH value for MP7 suggests strong molecular interactions and a more compact structure, requiring more incredible thermal energy for unfolding. This could be due to hydrogen bonding and hydrophobic interactions between MP and polyphenols at pH 7, stabilizing the protein structure [73]. In contrast, the lower ΔH value for MP9 suggests a more flexible or partially denatured structure at alkaline pH, likely due to reduced charge repulsion and increased aggregation.

Similarly, PP9 displayed a high enthalpy change, suggesting strong protein–polyphenol interactions at pH 9. The increased enthalpy values at alkaline pH indicate that polyphenol binding induces structural reinforcement, possibly through protein cross-linking, which enhances thermal stability [74]. In contrast, SMP9 had a significantly lower ΔH, indicating that the structural integrity of SMP was less preserved under alkaline conditions, resulting in weaker molecular interactions.

The measurements showed that SMP and MP retained significant thermal resistance under these conditions. SMP9 had the greatest T_max_, followed by SMP3 and MP9. Because polyphenol binding stabilizes the proteins’ tertiary and secondary structures, the high T_max_ values indicate increased protein stiffness. Alternatively, MP7 had the lowest T_max_, suggesting that MP was less stable structurally at neutral pH, possibly due to unfolding interactions that facilitated complex formation but decreased resistance to heat denaturation [69].

With PP9 displaying higher thermal stability than PP3, the PP samples showed intermediate T_max_ values, indicating that polyphenol–protein interactions at alkaline pH contribute to thermal reinforcement. These results are consistent with the study by Muntaha et al. (2025) [9], which found that polyphenols can enhance the heat stability of proteins by reducing protein unfolding and strengthening intra- and intermolecular interactions.

SMP9 and PP7 showed the greatest T_end_, indicating extended stability during thermal processing. The idea that MP experiences more structural unfolding at neutral pH is further supported by the fact that MP7 had the lowest T_end_. According to the observed trends, proteins interacting with polyphenols at alkaline pH typically show higher T_max_ and T_end_ values, indicating better thermal stability. However, depending on their molecular composition and affinity for binding polyphenols, various proteins exhibit different levels of stability. Overall, pH, interactions between proteins and polyphenols, and molecular conformational changes all significantly impacted the thermal stability of protein–polyphenol complexes.

### 3.12. FT-IR Evaluation

**Figure 9:**
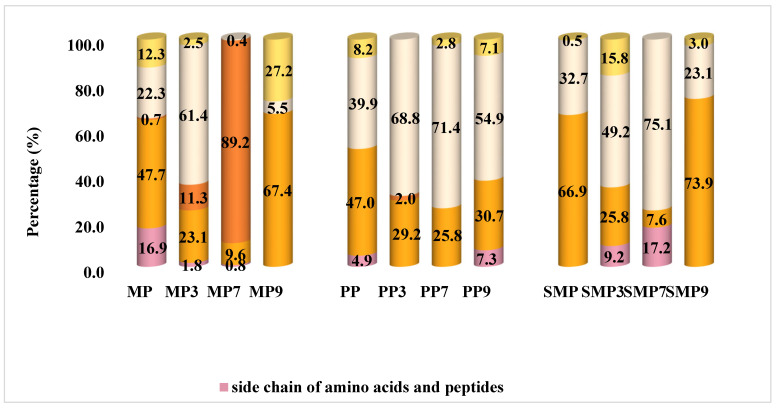
Secondary structure composition of protein isolates and their complexes by FT-IR.

The secondary structure composition of protein isolates and their polyphenol complexes was analyzed by FT-IR spectroscopy, with the results presented in Figure 9.

FT-IR spectra revealed distinct structural modifications in protein isolates upon interaction with anthocyanin. MP, PP, and SMP exhibited a significant shift in secondary structure composition, particularly in the contents of β-sheets and α-helices. The β-sheet proportion was notably high in MP7, MP9, and SMP9, suggesting a strong interaction between mustard and sunflower meal proteins with polyphenols at neutral and alkaline pH. This could be due to the increased availability of interaction sites at these pH levels or the specific affinity of polyphenols for certain amino acid residues exposed under these conditions [55].

Significant structural changes were also observed across pH levels in PP samples. With a substantial percentage of α-helix structures and a high β-sheet content, PP3 showed a relatively stable conformation. This stability may be due to the balanced contribution of these secondary structures, which provides a robust structural framework [69]. The most significant β-sheet proportion and lowest random coil content were found in PP7 at neutral pH, indicating improved molecular ordering, most likely due to strong hydrogen bonds between polyphenols and protein backbones [75]. On the other hand, PP9 exhibited an increase in random coils and a shift towards β-turn structures, indicating enhanced structural flexibility at alkaline pH. According to these results, PP retains a stable structure at neutral pH, but at alkaline pH, enhanced polyphenol interactions encourage structural loosening, possibly due to electrostatic repulsion or steric hindrance. This flexibility could impact the material’s functional characteristics, such as solubility, emulsifying properties, or digestibility [55].

SMP samples displayed relatively stable secondary structures across pH levels. SMP in its native state had a high β-sheet proportion, which remained unchanged across pH conditions. This inherent stability may be due to the amino acid composition or stabilizing intramolecular interactions within the protein [76]. SMP7 exhibited the highest β-sheet content and the lowest random coil proportion, suggesting that pH 7 enhances protein structural rigidity [35]. However, SMP9 showed a slight reduction in β-sheet content and an increase in β-turns, indicating some degree of structural rearrangement. Compared to MP and PP, SMP demonstrated the highest structural integrity upon polyphenol complexation, suggesting it is more resistant to pH-induced denaturation or aggregation.

Several molecular interactions contribute to these variations in secondary structure. Hydrogen bonding and π-π stacking interactions between polyphenols and protein residues likely stabilized β-sheet structures in MP9, PP7, and SMP7 [69]. Furthermore, the increase in β-sheet content in samples PP7 and SMP7 suggests that polyphenol binding promotes ordered protein aggregation, reinforcing structural stability. The results showed that the secondary structure composition of protein–polyphenol complexes is highly dependent on pH and polyphenol interactions, resulting in significant conformational changes that affect their functionality.

### 3.13. Microstructural Characterization by CLSM

**Figure 10:**
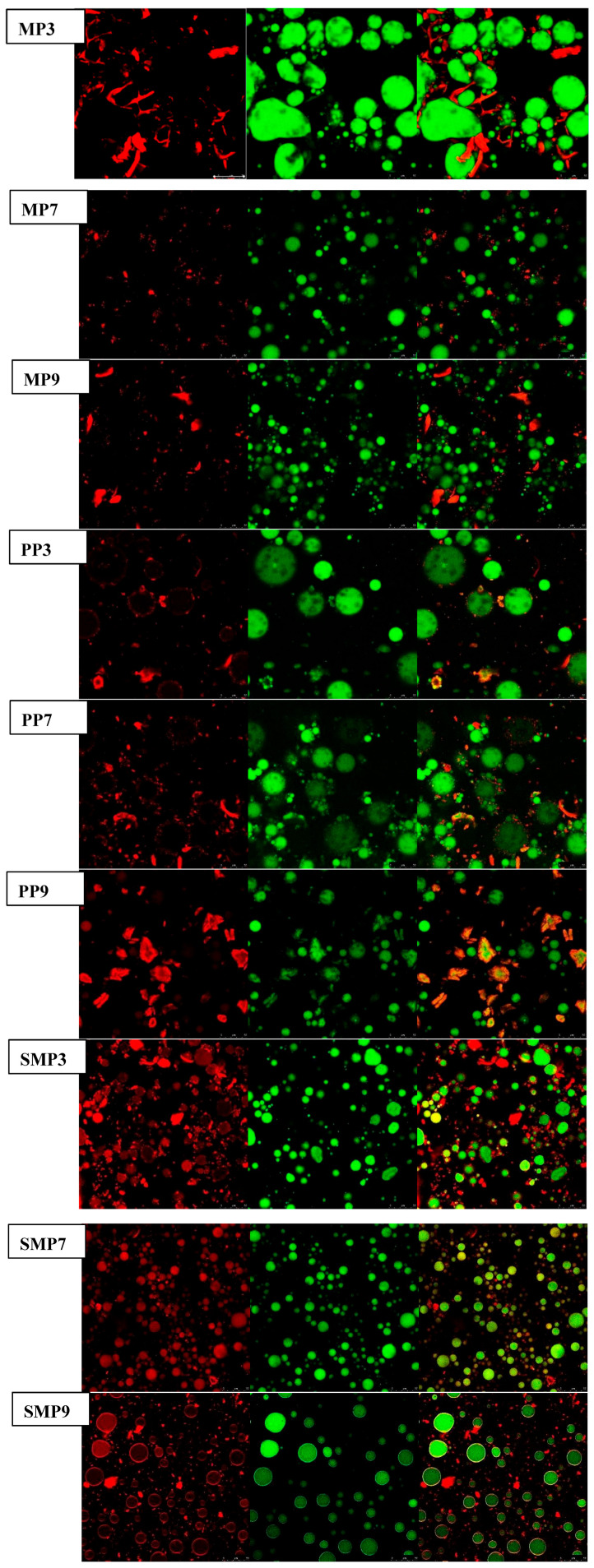
Confocal microscopy images of protein–polyphenol complex emulsions. Protein was stained red, and oil was stained green. The scale was 50 μm.

CLSM revealed important pH-dependent structural differences in protein–polyphenol complexes, especially in protein distribution (red) and oil droplets (green) [77]. MP3 had a highly aggregated protein network with dense, red fluorescent areas connected throughout the emulsion matrix, as indicated by the CLSM images. These findings imply substantial protein–polyphenol interactions at acidic pH, possibly (Figure 10). Due to electrostatic attractions between positively charged protein residues and polyphenols. The appearance of large, densely packed oil droplets in MP3 suggests that proteins were evenly distributed along the interface, which helped to stabilize the emulsion [75]. Nevertheless, MP7 exhibited a more uniform protein dispersion with reduced aggregation as the pH increased, indicating a shift in protein–polyphenol interactions that favored hydrogen bonding and hydrophobic processes. MP9 showed bigger oil droplets and disordered protein networks at alkaline pH, indicating weaker interfacial stability, possibly due to protein unfolding and reduced electrostatic interactions [35, 55].

A similar pattern was observed with PP samples. PP3 has a well-structured interfacial layer with evenly distributed protein networks, indicating high stability at acidic pH. At pH 7, PP7 had a more dispersed protein structure and smaller oil droplets, indicating a higher emulsifying capacity. However, at pH 9, PP9 exhibited substantial protein aggregation, with visible protein clusters detaching from the oil droplets, indicating poor emulsion stability due to strong polyphenol–protein interactions that resulted in precipitation. These findings align with those of Xue, Feng et al. (2024) [78], who demonstrated that polyphenol-induced protein aggregation at alkaline pH can lead to phase separation and impaired emulsification efficiency.

Compared to MP and PP, SMP was more resistant to structural disruption. SMP3 showed small, well-dispersed oil droplets with a thick protein layer surrounding them, indicating high interfacial adsorption. This supports prior research that sunflower meal protein has superior emulsifying characteristics due to its amphiphilic nature [79]. At pH 7, SMP7 had a stable structure with low protein aggregation, but SMP9 showed minor protein separation from the oil phase, indicating mild instability but better structural retention than MP9 and PP9.

## 4. Conclusions

This study demonstrates that pH critically influences the structural and functional properties of protein–polyphenol complexes formed between mustard, primrose, and sunflower meal proteins with red cabbage-derived polyphenols. The findings reveal that acidic and alkaline conditions drive distinct structural rearrangements—such as changes in β-sheet content, surface hydrophobicity, and protein flexibility that modulate emulsifying capacity and antioxidant performance. Among the tested proteins, mustard and primrose showed greater pH sensitivity, offering higher emulsification or antioxidant responses under specific pH conditions, while sunflower protein exhibited more balanced stability across the pH range. These insights are valuable for designing clean-label functional ingredients tailored to pH-dependent food formulations, such as dressings, beverages, or nutraceutical emulsions. However, this work is limited by the absence of detailed compositional profiling of extracted polyphenols and by the lack of bioavailability or in vivo digestive studies. Future research should focus on the gastrointestinal stability of these complexes, their behavior in real food systems, and potential synergistic effects with other bioactive compounds.

## Figures and Tables

**Table 1:** Antioxidant activities (ABTS & DPPH), Enthalpy (ΔH), and thermal properties (T_on,_ T_max,_ and T_end_) of protein–polyphenol complexes.

Samples	ABTS (umol trolox/g)	DPPH (umol trolox/g)	Enthalphy (ΔH–J⋅g^−1^)	T_on_ [°C]	T_max_ [°C]	T_end_ [°C]
MP3	59.35 ± 1.47 ^c^	115.58 ± 0.15 ^a^	−121.90	157.56	161.17	175.42
MP7	28.71 ± 0.46 ^a^	86.69 ± 0.15 ^a^	−257.80	56.11	105.83	151.02
MP9	46.77 ± 2.28 ^b^	112.69 ± 1.71 ^a^	−98.40	163.68	166.33	177.67
PP3	54.83 ± 2.74 ^bc^	358.31 ± 0.15 ^b^	−158.00	147.58	151.33	171.58
PP7	114.82 ± 0.91 ^d^	635.71 ± 0.15 ^c^	−173.30	152.28	160.50	194.30
PP9	377.36 ± 1.82 ^f^	1248.30 ± 0.45 ^c^	−215.40	154.58	158.50	176.48
SMP3	9.68 ± 2.74 ^e^	132.92 ± 0.45 ^a^	−122.80	164.55	167.83	180.47
SMP7	34.51 ± 2.11 ^a^	340.97 ± 0.75 ^b^	−154.90	152.54	155.17	167.74
SMP9	109.98 ± 2.28 ^d^	118.47 ± 1.40 ^a^	−111.00	138.71	168.00	192.27

Data are represented as mean ± SD; different superscript letters indicate statistical significance at *p* ≤0.05. Thermal parameters include the enthalpy change (ΔH), onset temperature (Ton), maximum denaturation temperature (Tmax), and endset temperature (Tend) obtained from the DSC thermograms.

## Data Availability

The original contributions presented in this study are included in the article. Further inquiries can be directed to the corresponding author.
